# Prevalence of risk factors for stuttering among boys: analytical cross-sectional study

**DOI:** 10.1590/1516-3180.2014.1323617

**Published:** 2014-04-14

**Authors:** Cristiane Moço Canhetti Oliveira, Paula Roberta Nogueira

**Affiliations:** I PhD. Professor, Postgraduate Program on Speech and Hearing Disorders, Department of Speech and Hearing Disorders, Universidade Estadual Paulista (Unesp), Marília, São Paulo, Brazil; II Speech Therapy Student, Department of Speech and Hearing Disorders, Universidade Estadual Paulista (Unesp), Marília, São Paulo, Brazil

**Keywords:** Stuttering, Risk factors, Speech disorders, Genetics, Speech, language and hearing sciences, Gagueira, Fatores de risco, Distúrbios da fala, Genética, Fonoaudiologia

## Abstract

**CONTEXT AND OBJECTIVE:**

There have been few studies on the risk factors for subgroups of stuttering. The aim of this study was to characterize the risk factors for developmental familial stuttering among boys who stutter and who do not stutter, such as disfluency types, associated quality and communication factors, emotional and physical stress, familial attitudes and personal reactions.

**DESIGN AND SETTING:**

Analytical cross-sectional study with a control group, performed at the Fluency Studies Laboratory of the Department of Speech and Hearing Disorders of a public university.

**METHODS:**

The parents of 40 age-matched stuttering and non-stuttering boys took part in this study. The participants were divided into two groups: stuttering children (SC) and non-stuttering children (NSC), with ages between 6 years 0 months and 11 years 11 months. Initially, all of the participants underwent a fluency assessment and then data were gathered using the Protocol for the Risk of Developmental Stuttering.

**RESULTS:**

There were no differences in the physical stress distribution factors and personal reactions between the groups. Inappropriate familial attitudes were presented by 95% of the SC and 30% of the NSC. Four risk factors analyzed were not shown by the NSC, namely stuttering-like disfluency, quality factors, physical stress and emotional stresses.

**CONCLUSIONS:**

The findings suggest that the presence of stuttering-like disfluency, associated quality and communication factors, emotional stress and inappropriate family attitudes are important risk factors for familial developmental stuttering among boys.

## Abstract (pt)

**CONTEXTO E OBJETIVO:**

Há poucos estudos sobre os fatores de risco para os subgrupos de gagueira. O objetivo deste estudo foi caracterizar os fatores de risco para a gagueira desenvolvimental familial em meninos que gaguejam e que não gaguejam como tipologia das disfluências, fatores qualitativos e comunicativos associados, estresse físico e emocional, atitude familiar e reação pessoal.

**TIPO DE ESTUDO E LOCAL:**

Estudo transversal analítico com um grupo controle, realizado no Laboratório de Estudo da Fluência, que faz parte do Departamento de Fonoaudiologia de uma universidade pública.

**MÉTODOS:**

Pais de 40 meninos com e sem gagueira pareados por idade fizeram parte do estudo. Os participantes foram divididos em dois grupos: crianças com gagueira e crianças sem gagueira, com idades entre 6 anos 0 meses e 11 anos 11 meses Inicialmente todos os participantes foram submetidos a uma avaliação de fluência e depois os dados foram coletados por meio do Protocolo de Risco para a Gagueira do Desenvolvimento.

**RESULTADOS:**

Não foi observada diferença nos fatores de estresse físicos e reações pessoais entre os grupos. As atitudes inadequadas familiares foram apresentadas por 95% das crianças com gagueira e 30% das crianças sem gagueira. Quatro fatores de risco analisados não ocorreram nas crianças sem gagueira, a saber, disfluências gagas, fatores qualitativos, estresse físico e emocional.

**CONCLUSÕES:**

Os achados sugerem que a presença de disfluências gagas, fatores qualitativos e comunicativos associados, estresse emocional e atitude familiar inadequada são fatores de risco importantes para a gagueira desenvolvimental familial em meninos.

## INTRODUCTION

Stuttering is a multifactorial and complex disorder that results from the influence of many factors, which include genetic predisposition, motor speech skills, linguistic skills and cognitive, emotional and environmental factors.^1^ It is know that the spectrum of risk factors for stuttering is wide and heterogeneous.^2^ Stuttering typically begins during the preschool years, which suggests that many important factors act during this developmental process.^3^

There is a clinically important reason for identifying stuttering among preschoolers. It has been shown that young stuttering children respond well to direct intervention, thereby helping to prevent the disorder from developing into a more intractable chronic form.^4^ ^,^ ^5^ Therefore, early diagnosis and intervention are important and, for this reason, the risk factors for persistent developmental stuttering need to be investigated.^6^ ^-^ ^8^

A wide range of possible risk factors has been proposed in the literature, including age; gender; type and manner of onset; duration of the disfluency; type of disfluency; associated communicative and qualitative factors; physical and emotional stress; family history of stuttering; personal, familial and social reaction; and family attitudes.^9^

Gender is an important risk factor for stuttering, given that stuttering is more prevalent among males.^3^ ^,^ ^10^ ^,^ ^11^ This risk among boys is higher when any communication disorder is present, independent of the family history.^8^

Associated qualitative factors, such as body and facial muscle tension, rapid speech rate, uncoordinated breathing and/or vocal intensity and vocal frequency variation may be present together with the disfluency seen among children who stutter.^12^

Regarding physical stress, some authors^11^ ^,^ ^13^ have reported that the origin of sporadic stuttering (without a family history of stuttering) may be found in perinatal or childhood physical trauma that perhaps caused some brain dysfunction. Some examples of the physical stress that occurs just prior to the onset of stuttering have included such conditions as respiratory problems, surgery or illness requiring hospitalization, asthma requiring medical treatment, and acute illness.^3^

In the same way, emotional stress can contribute towards the beginning of stuttering, such as divorcing of parents, moving, death of a beloved pet, birthdays, family vacations or excessive sibling rivalry.^3^

Other communicative disorders can occur together with persistent developmental stuttering, like phonological or myofunctional disorders. Moreover, some studies have shown higher scores in vocabulary tests associated with stuttering beginning in young children.^3^ ^,^ ^14^

The disfluency of stuttering consists of repetition of sounds or syllables and blockage or prolongation of sounds.^15^ ^,^ ^16^

Several studies over past decades have shown that genetic factors are involved in transmission of susceptibility to stuttering.^17^ ^,^ ^18^ A positive family history may play an important role in the diagnosis process, since it increases the risk of persistent developmental stuttering.^3^ ^,^ ^6^

Family attitudes and inappropriate behavior regarding childhood disfluency can also have an influence through increasing the disruptions exhibited by such children.^19^ ^-^ ^21^ Another important point to be considered in this risk factor analysis is the personal reaction. People who are perfectionists or anxious, shy and insecure are more likely to suffer from persistent stuttering when it is associated with other risk factors.^9^

There have been few studies on the risk factors for subgroups of stuttering. Thus, it is very important to identify risk factors for stuttering and make comparisons with controls in order to check which are the primary risk factors for the disorder. Knowledge of these factors is important in adopting preventive measures and more appropriate treatment for stuttering.

## OBJECTIVE

The aim of this study was to characterize the risk factors for developmental familial stuttering among boys who stutter and who do not stutter, such as the type of disfluency, associated quality and communication factors, emotional and physical stress factors, family attitudes and personal reactions. 

## METHODS

This was an analytical cross-sectional study with a control group that was conducted at the Fluency Studies Laboratory of the Department of the Speech and Hearing Disorders of a public university clinic. This study included boys who stuttered with ages between 6 years 0 months and 11 years 11 months. The present study used a non-probabilistic convenience sample that originally comprised 45 stutterers who were seen at the Fluency Studies Laboratory between April 2012 and September 2012, of whom 36 were children between 6 years 0 months and 11 years 11 months of age, but 9 were girls. Thus, the group was initially composed of 27 boys who stuttered. However, only 20 of them met the inclusion criteria. 

The procedure for selecting the 20 boys who did not stutter consisted of recruitment among the students at a public school who met the inclusion criteria. In this manner, the study sample was composed of 40 boys aged 6 years 0 months and 11 years 11 months, of whom 20 were stutterers and 20 were non-stutterers.

The protocol for this study was approved by the institution's Ethics Committee (n^o^ 0396/2011), and the adults responsible for these children signed a consent statement. 

### Subjects 

Forty age-matched stuttering and non-stuttering children and their parents took part in this study. The participants who stuttered were invited when attending the Fluency Studies Laboratory, which is part of the speech clinic at a public university. Fluent control participants were recruited through contacting students at a public school in Marília, Brazil, the city where the study was developed.

The participants were divided into two groups: stuttering children (SC) and non-stuttering children (NSC), in order to investigate whether there were any differences in relation to any of the risk factors for persistent developmental stuttering that were analyzed. 

The SC group was composed of 20 boys with ages between 6 years 0 months and 11 years 11 months, with a diagnosis of stuttering. The NSC group was composed of 20 boys who were age-matched with the SC.

The inclusion criteria for the two groups were as follows: age between 6 years 0 months and 11 years 11 months, male gender and speakers of Brazilian Portuguese, without any other associated communication, neurological, hearing, visual or cognitive shortfall.

The inclusion criteria for the SC group were as follows: stuttering disorders reported by both parents; developmental stuttering present before 10 years of age; minimum duration of disfluency of 12 months, without remission (persistent); demonstration of stuttering in at least 3% of the syllables in the speech sample obtained by the examiner;^22^ ^-^ ^24^ score of 11 points or more (i.e. severity equivalent to at least "mild") on the Stuttering Severity Instrument 3 (SSI-3).^25^

The selection criterion for the NSC were as follows: no personal or family history of stuttering and/or cluttering; presentation of not more than two instances of stuttering-like disfluency per 100 syllables of conversational speech; and a total overall score of 10 points or lower (i.e. a severity equivalent of less than "mild") on the Stuttering Severity Instrument 3 (SSI-3).^25^

### Procedures 

Initially, all of the boys underwent a fluency assessment to separate them into the two groups (SC and NSC) and then data were gathered using the Protocol for the Risk of Developmental Stuttering (PRGD).^26^

Speech samples were obtained in situations of spontaneous speech. Each speech sample was audiotaped and contained at least 200 fluent syllables. The samples were transcribed literally.^26^ The types of disfluency were analyzed to differentiate between other disfluencies (OD) and stuttering-like disfluencies (SLD), in accordance with the Fluency Profile Assessment,^26^ and thus to calculate the percentage of stuttered syllables. 

The stuttering severity was determined by means of an international instrument (the Stuttering Severity Instrument, SSI-3).^25^ This test assesses the frequency and duration of speech disruptions, and also the presence of physical concomitants associated with these disruptions. Based on these parameters, the stuttering severity index was determined as very mild, mild, moderate, severe or very severe.

Any histories of important risk factors for persistent developmental stuttering, like age, type of disfluency, associated communicative and qualitative factors, physical and emotional stress, family history, family attitudes and personal reactions (Protocol for the Risk of Developmental Stuttering, PRGD)^9^ were elicited from all the participants' parents (SC and NSC groups). 

### Statistical analysis 

The results were expressed as percentages of the presence of each risk factor, analyzed for the two groups. The P values were calculated by means of the chi-square test. Descriptive values below 5% (P value < 0.05) were considered statistically significant. The data were analyzed using the Statistical Package for the So cial Sciences (SPSS) 20.0 software (SPSS Inc., Chicago, USA).

## RESULTS

With regard to the purpose of this study, the data obtained are presented in table form. Table 1 shows the individuals' ages, the total number of stuttering-like disfluencies (SLDs), the percentage of stuttered syllables (%ss) and the total score of the Stuttering Severity Instrument (SSI-3).^25^

**Table 1.** Means and standard deviations (SD) of age and speech fluency results of the stuttering children (SC, n = 20) versus non-stuttering children (NSC, n = 20)

	SCMean (SD)	NSCMean (SD)
Age	7.50 (1.43)	7.55 (1.43)
Stuttering-like disfluency	14.0 (8.80)	2.00 (1.84)
Percentage of stuttered syllables	7.00 (4.40)	1.00 (0.92)
**Total score for Stuttering Severity Instrument**	**23.0 (6.87)**	**6.00 (1.10)**

Table 2 describes the risk factors for persistent developmental stuttering among the SC and NSC. There were no differences in the distribution of physical stress or personal reactions between the groups. Inappropriate family attitudes were shown by 95% of the SC and 30% of the NSC. Four risk factors that were analyzed were not shown by the NSC, i.e. stuttering-like disfluency, quality factors (rapid speech rate, uncoordinated breathing or associated stress), communication factors (phonological or myofunctional disorders) and emotional stress (parental death, parental divorce or familial or parental disease or illness).

**Table 2.** Prevalence of risk factors among stuttering and non-stuttering children, with P-values

Risk factors	Stuttering children	Non-stuttering children	P-value
Stuttering-like disfluencies	100%	0%	< 0.001
Quality factors	100%	0%	< 0.001
Communicative factors	75%	0%	< 0.001
Physical stress factors	35%	10%	0.058
Emotional stress factors	50%	0%	< 0.001
Family attitudes	95%	30%	< 0.001
Personal reactions	95%	100%	0.0311

## DISCUSSION

Several studies have shown that, in cases of stuttering, the earlier an intervention is instituted, the more favorable the outcome is.^4^ ^,^ ^5^ Therefore, studying the risk factors for this disorder is very important for clinical practice and for improving the knowledge of developmental stuttering. Our study showed the importance of studying risk factors for developmental stuttering in Brazil.^8^ ^,^ ^27^ ^,^ ^28^

The present study on the risk factors for persistent developmental stuttering forms an important part of the process of diagnosing fluency disorders. These data, together with fluency assessment data will lead to precise diagnosis and support for the therapeutic process.

In this study, some significant factors were associated with familial persistent developmental stuttering among boys, such as stuttering-like disfluency (SLD) and quality, communication and emotional stress factors, as well as family attitudes. This suggests that occurrences of these factors are associated with a higher risk of stuttering for this subgroup. Furthermore, the results showed that there were no differences between the groups (SC versus NSC) with regard to physical stress factors and personal reactions.

Regarding stuttering-like disfluencies (SLDs; i.e. sound-syllable repetition, prolongation and blockage), our study confirmed that observable speech disruption is a central feature of this disorder.^29^ Thus, this is considered to be a chronic disorder that involves involuntary disruptions in fluent speech.^30^ Developmental stuttering presents as a chronic disruption of an individual's ability to produce smooth, effortless and forward-moving speech.^31^ In other words, the principal manifestation of the stuttering, regardless of gender, is stuttering-like disfluencies (SLDs). Therefore, this is not specifically a characteristic of the subgroup of boys.

All the children who stuttered presented at least one of the quality factors, while all the children who did not stutter did not present these, according to their parents' reports. A previous study correlated the associated quality factors, such as body and facial muscle tension, rapid speech rate, uncoordinated breathing and/or vocal intensity and vocal frequency variation, with disfluencies among SC.^9^ In the present study, we confirmed that disfluencies among SC can be followed by quality factors.

The majority of our children who stuttered presented some communication factors, and all the children who did not stutter did not present these, according to their parents' reports. These results are in accordance with another study that reported that children who stuttered frequently presented other associated communication factors such as phonological or myofunctional disorders.^9^

The present study confirmed the findings previously published regarding speakers of Brazilian Portuguese, in relation to emotional stress factors. This previous study also found a significant relationship between persistent developmental stuttering among boys and emotional stress factors.^28^ Our study presents evidence that stuttering is associated with multiple factors, like many other authors have previously shown.^1^ ^,^ ^2^

In our study, we found that family attitudes were inappropriate in the cases of 95% of the children who stuttered and 30% of the controls. Therefore, this finding confirmed that the prevalence of inappropriate family attitudes among children who stuttered was higher than among the controls. In another cross-sectional study carried out in São Paulo, Brazil, it was found that the highest risk factor for worse stuttering was the quality of the parents' behavior, with significant differences.^9^ Like in the present study, other authors have also demonstrated that among the families of children who stuttered, inappropriate family attitudes were commonly found.^9^ ^,^ ^19^ For example, faster speaking rates among mothers were associated with greater stuttering severity in their children.^21^

We also found that in this subgroup of persistent developmental stuttering, there was no difference between the SC and NSC in relation to physical stress factors. This finding is similar to that of another study on Brazilian children with familial stuttering, which showed no relationship between their stuttering and physical stress factors.^28^ This result is also in line with the findings of Poulos and Webster,^13^ in which physical stress factors were correlated with the subgroup of sporadic developmental stuttering, i.e. individuals without any positive family history of stuttering. 

With regard to personal reactions, there was no significant difference between SC and NSC. This finding suggests, like in other studies, that personal reactions should not be considered to be a causal factor for development of stuttering.^32^ ^,^ ^33^ Thus, when the population consists of children, these reactions are not always clear. Although there are many studies on personality and temperament among people who stutter,^34^ ^-^ ^37^ there is still no consensus on whether stuttering indeed has any significant impact on people's personalities. One reason for the contradictory results obtained in this field is the lack of use of standardized questionnaires.^38^ These results corroborate other studies and will be an essential tool in designing and implementing future clinical protocols for diagnosing childhood stuttering in order to determine the risk of this disorder that children present. 

In order to critically evaluate this study, it is necessary to consider the limitations inherent to the research design and analysis of the study. Firstly, the method of parental reporting that was used to study the risk factors has certain limitations. Although the parents' answers were restricted in an attempt to gather unambiguous responses, the reporting remains the result of the parents' perceptions, sensitivity, memory and interpretation, thereby adding some variance to the data. The presence of the researcher during the face-to-face interviews may have influenced the information given by the parents, since they may have provided answers that they thought the researcher wished to hear. Secondly, all the participants were students at a public school and thus were of lower socioeconomic level. Lastly, the findings from this study are subtle and based on a subgroup of developmental stuttering, i.e. familial persistent developmental stuttering among boys, and hence the ability to generalize these results to the wider population of children who stutter may be limited.

These limitations, as well as the findings from the current study, suggest important areas for future research. For example, replication of this type of study among girls who stutter might elicit additional data with regard to gender differences among children who stutter. Furthermore, because the participants were children at a public school, it was not possible to explore different sociocultural factors. Therefore, another suggestion for future research is to study the risk factors in two groups: one composed of children from public schools and another of children from private schools.

## CONCLUSION

The findings suggest that the presence of stuttering-like disfluencies, quality and communication factors, emotional stress factors and inappropriate family attitudes are important risk factors for familial developmental stuttering among boys. Identifying these risk factors might make it possible to define the cases that require intervention, and thus, to provide earlier therapy.
